# Curcumin inhibits cerebral ischaemia–reperfusion injury and cell apoptosis in rats through the ERK–CHOP–caspase-11 pathway

**DOI:** 10.1080/13880209.2022.2069271

**Published:** 2022-05-20

**Authors:** Yue Chen, Lixia Zhang, Zengtai Yang, Jie Yu

**Affiliations:** a Department of Pediatrics, The Center Hospital of Cangzhou, Cangzhou, China; b Department of Pediatrics, The Peoples Hospital of Hejian, Hejian, China; c Cardiology Department, The Peoples Hospital of Hejian, Hejian, China

**Keywords:** Glial cells, neuroinflammatory response, brain oedema, signal channel

## Abstract

**Context:**

Curcumin has a significant effect on cerebral ischaemia–reperfusion injury (CIRI). However, the underlying mechanism is less studied.

**Objective:**

This study investigates the role and mechanism of curcumin in CIRI.

**Materials and methods:**

CIRI model Sprague-Dawley rats were divided into model, positive control and curcumin low/middle/high dose (50, 100 and 200 mg/kg/d) groups (*n* = 10 each). Drug intervention was administered by gavage once a day for 4 weeks. We calculated the neurobehavioural score and observed the cerebral infarct volume. Glial cytopathological changes were observed after haematoxylin–eosin staining. Apoptosis was detected by TUNEL (TdT mediated dUTP nick end labelling). Extracellular signal-regulated protein kinase (*ERK*), C/EBP-homologous protein (*CHOP*) and *caspase-11* mRNA were detected by real-time PCR. Phosphorylated ERK (p-ERK), phosphorylated CHOP (p-CHOP) and caspase-11 were detected by Western blot. Superoxide dismutase (SOD) activity was detected by xanthine oxidation method; malondialdehyde (MDA) content by thiobarbituric acid colorimetry; and, glutathione (GSH) by spectrophotometry.

**Results:**

Compared with control, the neurobehavioural scores, neuronal apoptosis, MDA, IL-1β, IL-18, mRNAs and protein levels of ERK/p-ERK, CHOP/p-CHOP and caspase-11 in model group were significantly higher (*p* < 0.01). Compared with model, the positive control and medium/high dose curcumin groups were significantly lower (*p* < 0.01). However, SOD and GSH decreased significantly in model group but increased significantly in positive control and medium/high dose curcumin groups (*p* < 0.01). Moreover, curcumin significantly alleviated ischaemic state and neuroinflammation (*p* < 0.01).

**Discussion and conclusions:**

Curcumin may alleviate CIRI through ERK–CHOP–caspase-11 pathway. Our results may provide new insights into the pathogenesis of CIRI, and contribute to the development of treatment strategies for CIRI.

## Introduction

Cerebrovascular disease is one of the major diseases that seriously endanger human life and health. Ischaemic injury occurs in 60–80% of patients with cerebrovascular disease, and it mainly causes temporary or permanent slowdown of blood flow, which leads to structural and functional damage in large areas of brain tissue (Wu et al. 2018; Jin et al. 2019). In addition to the ischaemic injury, the damage caused by reperfusion is more serious (He et al. 2015). Cerebral ischaemia–reperfusion injury (CIRI) is accompanied by a series of complex pathophysiological mechanisms, such as excitotoxicity, oxidative stress, inflammation and apoptosis (Xing et al. 2012; Yang et al. 2017). Neuroinflammation is an important and complex pathophysiological process in cerebral ischaemia, which is involved in the process from early injury to tissue repair after ischaemia (Palencia et al. 2015). So far, the exact molecular signalling pathways involved in cerebral ischaemia have not been fully elucidated, leading to difficulties in clinical treatment.

The extracellular signal-regulated protein kinase (ERK) pathway is involved in the occurrence and development of cerebrovascular diseases, and the survival and proliferation of cells are usually regulated by the ERK pathway (Liang et al. 2018; Zhang et al. 2019). C/EBP-homologous protein (CHOP)–caspase-11 pathway may play an important role in mediating inflammatory response and inducing cell damage and death after cerebral ischaemia. For example, CHOP–caspase-11 induces the release of IL-1β and IL-18 in ischaemic reperfusion (Liu et al. 2018).

Curcumin is a natural effective ingredient extracted from the rhizomes of Araceae and Zingiberaceae. It has many pharmacological effects, such as anti-inflammation, choleretic effects, antibacterial infection, antitumor, lipid-lowering effects, antioxidation and immunomodulation (Chen et al. 2014). Studies have reported (Cheng et al. 2013; Zhu et al. 2015) that curcumin has a significant effect in the treatment of CIRI, can suppress neuroinflammation caused by cerebral ischaemia, and inhibit cell apoptosis. However, the underlying mechanism is less studied.

In this study, we investigate the effect of curcumin on CIRI. The possible mechanism involving the ERK–CHOP–caspase-11 pathway was also analysed and discussed.

## Materials and methods

### Animals

A total of 60 Sprague-Dawley rats (30 male rats and 30 female rats, weight 180–220 g) were purchased from the Experimental Animal Centre of Hebei College of Traditional Chinese Medicine (license number: SCXK (Hebei) 2019-0074). The rats were housed at a constant temperature (24 ± 1 °C) with a 12 h dark/light cycle. All rats had free access to food and water. All animal experimental procedures were approved by the Ethics Committee of the Centre Hospital of Cangzhou (approval no. ECCHCZ: 2019-101).

### Reagents and instruments

**Figure 1.**
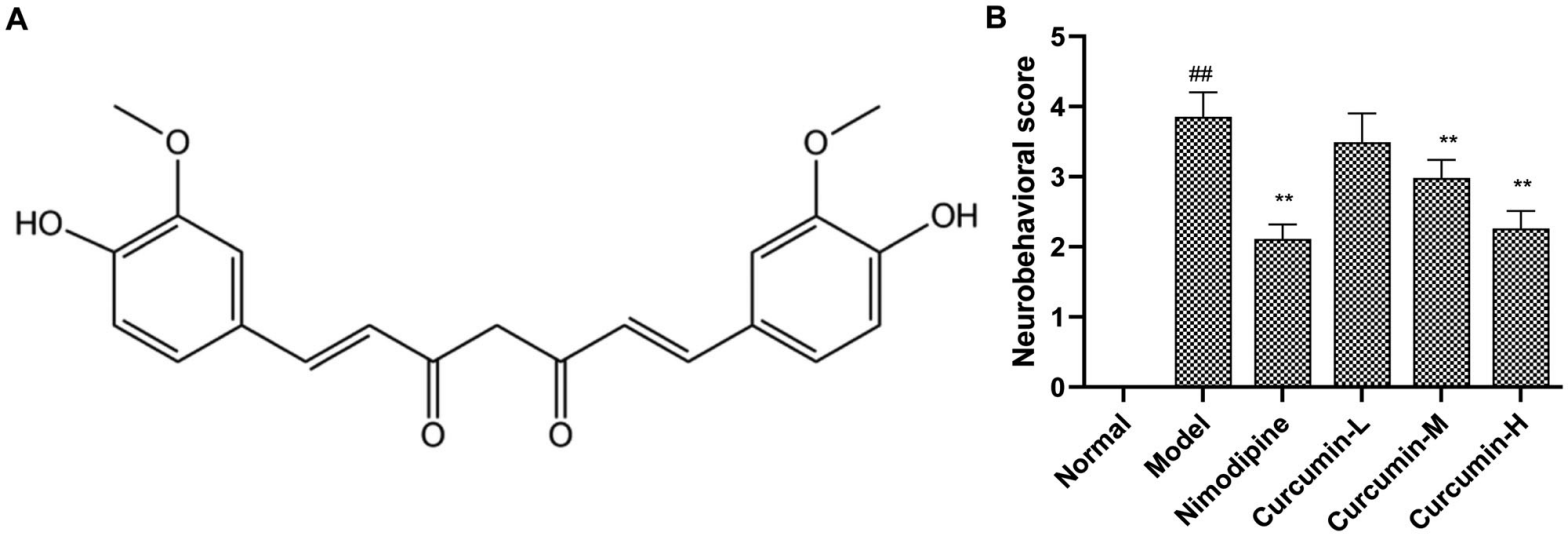
Effect of curcumin on neurobehavioural scores. (A) Chemical structure of curcumin. (B) Analysis of neurobehavioural scores. The neurobehavioural scores were evaluated based on a five-point scale. Compared with the normal, ^##^*p* < 0.01; compared with the model, **p < 0.01.

The details of the main reagents were as follows: curcumin (purity >97%; chemical structure in Figure 1(A); Hebei Tianxu Biological Technology Co., Ltd., Shijiazhuang, China); nimodipine (Yabao Pharmaceutical, Yuncheng, China); 4% paraformaldehyde (Chemical Reagent Factory, Guangzhou, China); haematoxylin–eosin (HE) staining kit (Solarbio Science Technology Co., Ltd., Beijing, China); TUNEL (TdT mediated dUTP nick end labelling) apoptosis detection kit (Merck, Kenilworth, NJ); superoxide dismutase (SOD) kit (Ruji Biotechnology Development Co., Ltd., Shanghai, China); malondialdehyde (MDA) (Beyotime Institute of Biotechnology, Beijing, China); glutathione (GSH) kit (Gimeng Industrial Co., Ltd., Shanghai, China); rat IL-1β kit (Keruimei Technology Co., Ltd., Beijing, China); rat IL-18 kit (Thermo Fisher Technology (China) Co., Ltd., Shanghai, China); ERK, CHOP and caspase-11 polyclonal antibodies (Santa Cruz Biotechnology, Santa Cruz, CA).

The main instruments included: alphaEaseFC greyscale analysis software (Alpha Innotech, San Leandro, CA); T100 real-time PCR instrument (Bohui Biotechnology Co., Ltd., Beijing, China); DYY-1C gel imaging system (Liuyi Instrument Factory, Beijing, China); microscope (Nikon, Tokyo, Japan).

### Animal model establishment

According to a previous study (Longa et al. 1989), the modified Longa method was used to prepare the rat model of focal middle cerebral artery ischaemia–reperfusion injury. Briefly, the rats were anaesthetized by intraperitoneal injection of 10% chloral hydrate (3.0 mL/kg). After anaesthesia, the left common carotid artery, external carotid artery and internal carotid artery were separated. The external carotid artery was ligated, and the distal end of the internal carotid artery was clamped. An incision was made at the distal end of the common carotid artery, and a 0.234 mm diameter fish line with a blunt tip was slowly inserted into the common carotid artery and internal carotid artery. After loosening the clamp, the middle cerebral artery was occluded. After occlusion for 2 h, reperfusion was performed for 24 h. The rats in the normal control group received sham operation without occlusion. If the rats have symptoms of neurological deficit and can survive for 24 h after modelling, the ischaemia–reperfusion injury model was considered successful. No animals died during the modelling process and after modelling.

### Animal grouping and treatment

The animals with CIRI were randomly divided into model group, positive control group and curcumin low, middle and high dose groups, with 10 rats in each group. The rats (*n* = 10) without CIRI but sham-operated served as normal control group. The rats with CIRI but without drug intervention served as the model group. The positive control group, which included rats with CIRI, was given nimodipine (0.4 mg/kg/d), and the curcumin low, medium and high dose groups were given curcumin (50, 100 and 200 mg/kg/d) by gavage once a day, continuously for 4 weeks. The normal control group and the model group were given equal volumes of normal saline. After 4 weeks of drug administration, the neurobehaviours of rats were evaluated and scored. Then, rats were anaesthetized with intraperitoneal injection of 10% chloral hydrate (3.0 mL/kg). Blood samples were collected from the heart. After that, rats were euthanized by cervical dislocation and brain tissues were collected.

### Neurobehavioural scoring

The neurobehaviours of rats in each group were scored on a five-point scale as previously described (Zhu et al. 2018). If there is no obvious functional loss, it is scored as 0; if the rats' contralateral front paw cannot be fully extended, one point; if the rats rotate to the opposite side when walking, two points; if the rats fall to the opposite side when walking, three points; if the rats are unable to walk spontaneously, and/or lost consciousness, four points.

### HE staining

The rat brain tissues were fixed in 4% paraformaldehyde, routinely dehydrated, embedded in paraffin and cut into 5 μm sections. The sections were subjected to HE staining with an HE staining kit according to the instructions.

### TUNEL assay

Cell apoptosis in CA1 area of hippocampus was assessed with TUNEL apoptosis detection kit according to the kit instructions. The apoptotic cells were stained brown-yellow. Five fields of view were randomly selected from each section. The number of apoptotic cells and the total number of cells were counted, and then the apoptosis index was calculated as follows:

Apoptosis index=number of apoptotic cells/(total number of cells)×100%.

### Detection of SOD, MDA, GSH content in rat brain tissue

The brain tissues were subjected to lysis with RIPA buffer. After centrifugation, the supernatant was collected and mixed with 0.9% sodium chloride solution at the ratio of 1:9 to make the brain tissue homogenate. After centrifugation at 4000 rpm for 10 min, the supernatant was collected. The SOD activity, MDA content and GSH content in the supernatant were detected with xanthine oxidation method, thiobarbituric acid method and spectrophotometric method, respectively, strictly following the instructions of the respective kits.

### ELISA

The serum samples were obtained after centrifugation of the blood samples at 4000 rpm for 10 min at 4 °C. The brain homogenate was prepared as above described. The IL-1β and IL-18 in serum and brain tissues were measured by ELISA according to the kit instructions. The concentrations were calculated according to the standard curve.

### Real-time PCR

The expression levels of *ERK*, *CHOP* and *caspase-11* mRNA in brain tissues after cerebral ischaemia and reperfusion were measured. Total RNAs were extracted from brain tissues with Trizol. The 1% agarose gel electrophoresis was used to identify the integrity of RNA, and the purity of RNA was detected at 260 nm. The primer sequences are shown in Table 1. The real-time PCR reaction conditions were as follows: 95 °C/15 s, 66 °C/20 s, 72 °C/30 s, 95 °C/15 s, 64 °C/20 s, 72 °C/30 s, 95 °C/15 s, 62 °C/20 s, 72 °C/30 s, 95 °C/15 s, 60 °C/20 s and 72 °C/30 s, with a total of 40 cycles. The gel density was scanned to calculate the relative level of *ERK*, *CHOP* and *caspase-11* mRNA.

**Table 1.** Primer sequences of real time PCR.

Gene	Primer	Sequences
β-actin	Forward	5′-TCT GGA AAG CTG TGC CGT G-3′
	Reverse	5′-CCA GTG AGC TTC CCG TTC AG-3′
ERK	Forward	5′-GUA CUU AAA UCG UGA AAC ACT CT-3′
	Reverse	5′-CTG TCA UGA AUU UAG CAC UUU GU-3′
CHOP	Forward	5′-GCC CAT GAC CAA CAT AAC TG-3′
	Reverse	5′-CCT TGA CGG CTA GTT GAT G-3′
Caspase-11	Forward	5′-AGG CAG AGA GGC AGG CAG AT-3′
	Reverse	5′-GGC GGG AGG TTT GAG ACA-3′

### Western blot

The proteins were extracted from brain tissues and the concentration of the protein was detected by the BCA method. The protein was electrophoresed on 12% SDS-PAGE gel and transferred to the membrane, which was then incubated with the primary antibodies of phosphorylated ERK (p-ERK) (1:1000), phosphorylated CHOP (p-CHOP) (1:500), caspase-11 (1:1000) and β-actin (1: 2000). β-actin was used as an internal control. After colour development with chemiluminescence, Quantity One software was used for greyscale analysis.

### Statistical analysis

SPSS 21.0 statistical software (SPSS Inc., Chicago, IL) was used to analyse the data. All data were expressed as mean ± standard deviation (SD). Multiple group comparisons were performed by ANOVA followed LSD *post hoc* test. A *p* < 0.05 was considered statistically significant.

## Results

### Effects of curcumin on neurobehavioural scores of rats in each group

Compared with the normal control group, the neurobehavioural scores of rats in the model group increased significantly (*p* < 0.01) (Figure 1(B)). Compared with the model group, the positive control group and the curcumin medium and high-dose groups had significantly reduced neurobehavioural scores (*p* < 0.01). The results indicate that curcumin improves the neurobehaviour of rats after CIRI.

### Effect of curcumin on the pathological changes of rat brain neurons in each group

**Figure 2.**
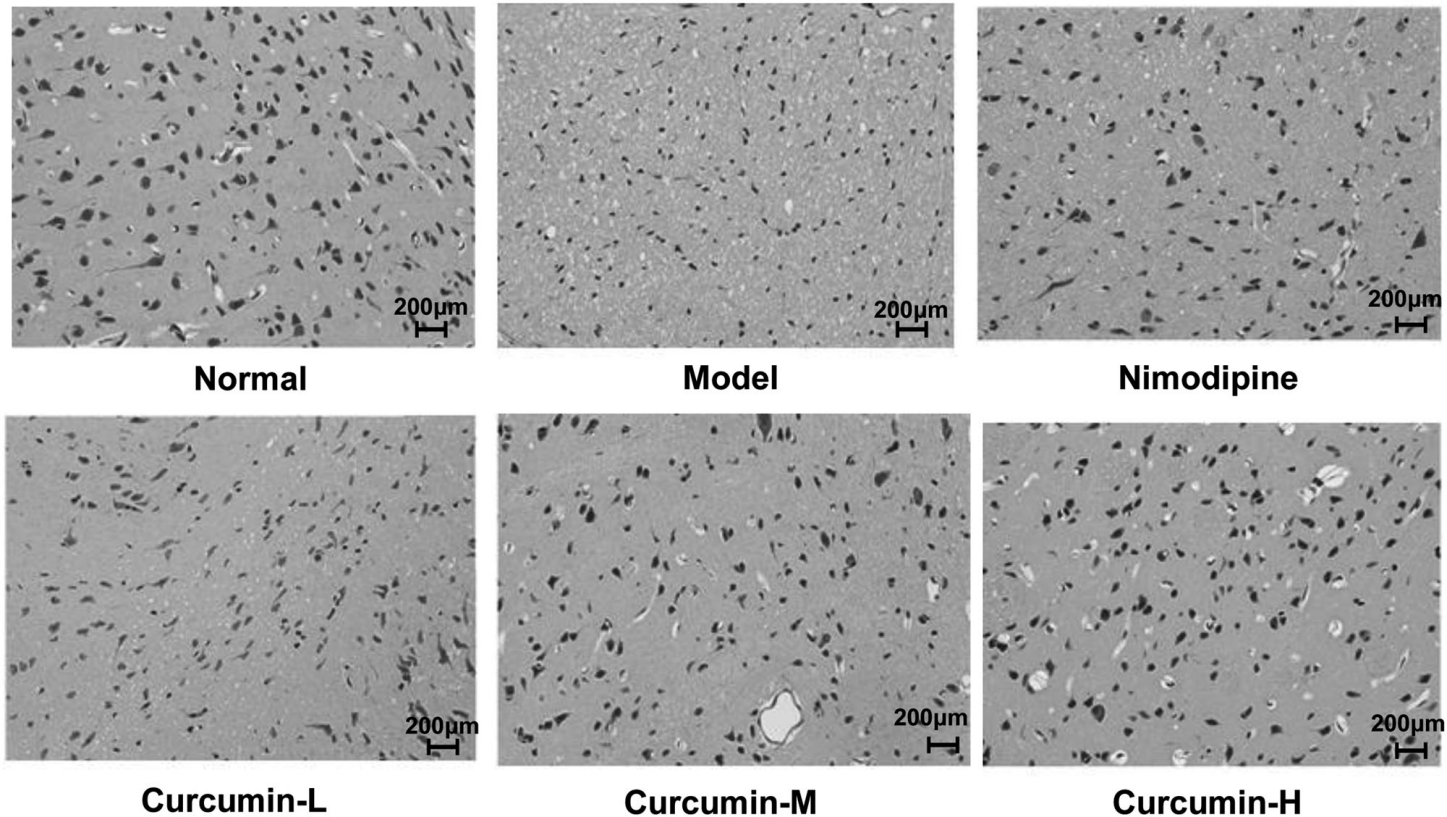
Effect of curcumin on glial cell pathology in rats. HE staining was performed. Representative images were shown. Scale bar: 200 μm.

HE staining was performed to observe the morphological changes of the brain neurons (Figure 2). The morphology of the brain neurons and glial cells of the rats in the normal control group was intact without abnormal changes. In the model group, the gap between brain tissues was enlarged. There was liquefaction and necrosis in the central area of the lesion. The neurons were severely shrunk. The lesion area of rats in the positive control group and the medium and high dose curcumin groups was reduced. The pathological state of the glial cells of the rats in the low dose curcumin group was basically the same as the model group. The results indicate that curcumin could minimize the pathological damage of rat glial cells caused by cerebral ischaemia and reperfusion.

### Effects of curcumin on neuronal apoptosis in hippocampal CA1 area of rats

**Figure 3.**
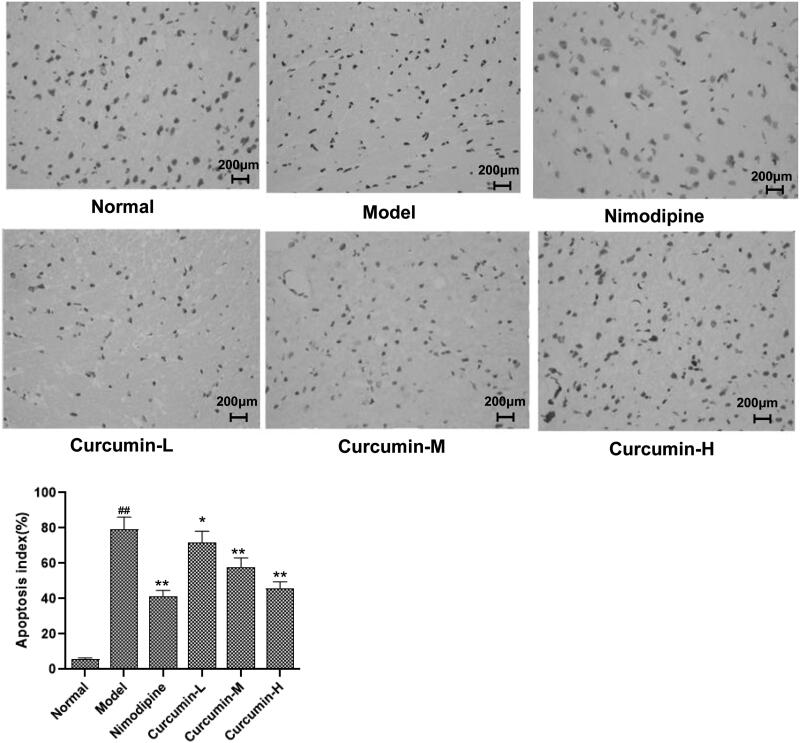
Effect of curcumin on neuronal apoptosis in hippocampal CA1 region of rats. TUNEL was used to assess apoptosis. Representative TUNEL staining images were shown. Apoptosis index was calculated as number of apoptotic cells/(total number of cells)×100%. Compared with the normal, ^##^*p* < 0.01; compared with the model, *p < 0.05, **p < 0.01. Scale bar: 200 μm.

To determine neuronal apoptosis, TUNEL assay was performed. As shown in Figure 3, there were occasionally irregular or apoptotic cells in the cortical area of the brain tissue of the normal control group. Compared with the normal control group, the number of apoptotic cells in the cortical area of the brain tissue of the model group increased significantly (*p* < 0.01); and compared with the model group, the number of neuronal apoptosis in the cerebral cortex of the positive control group and the middle and high-dose curcumin groups was significantly less (*p* < 0.01). Thus, curcumin alleviates neuronal apoptosis after cerebral ischaemia and reperfusion.

### Effects of curcumin on the contents of SOD, MDA and GSH in the brain tissues of rats

**Figure 4.**
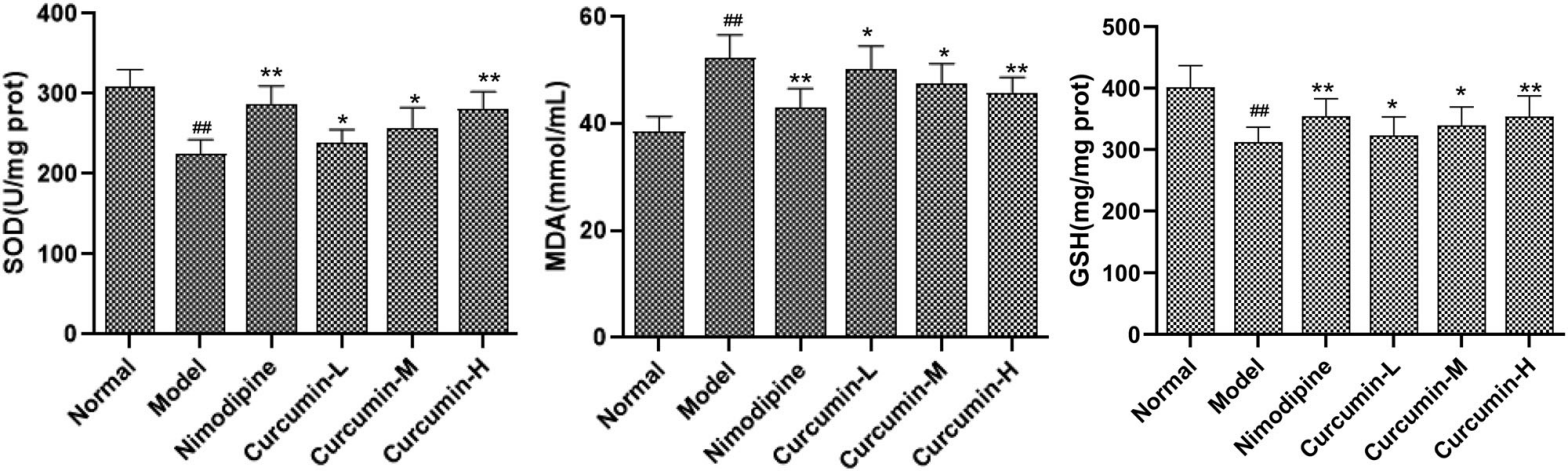
Effect of curcumin on SOD, MDA and GSH in brain tissues of rats. Compared with the normal, ^##^*p* < 0.01; compared with the model, *p < 0.05, **p < 0.01.

The contents of SOD, MDA and GSH in the brain tissues were measured. As shown in Figure 4, compared with the normal control group, the content of SOD and GSH in the brain tissue of the model group was significantly decreased, while that of MDA was significantly increased (*p* < 0.01). However, compared with the model group, the positive control group and the medium and high dose curcumin groups had significantly increased SOD and GSH while significantly decreased MDA (*p* < 0.01). Therefore, curcumin may reduce the oxidative stress after cerebral ischaemia and reperfusion.

### Effect of curcumin on the content of inflammatory factors IL-1β and IL-18 in serum and brain tissue of rats

**Figure 5.**
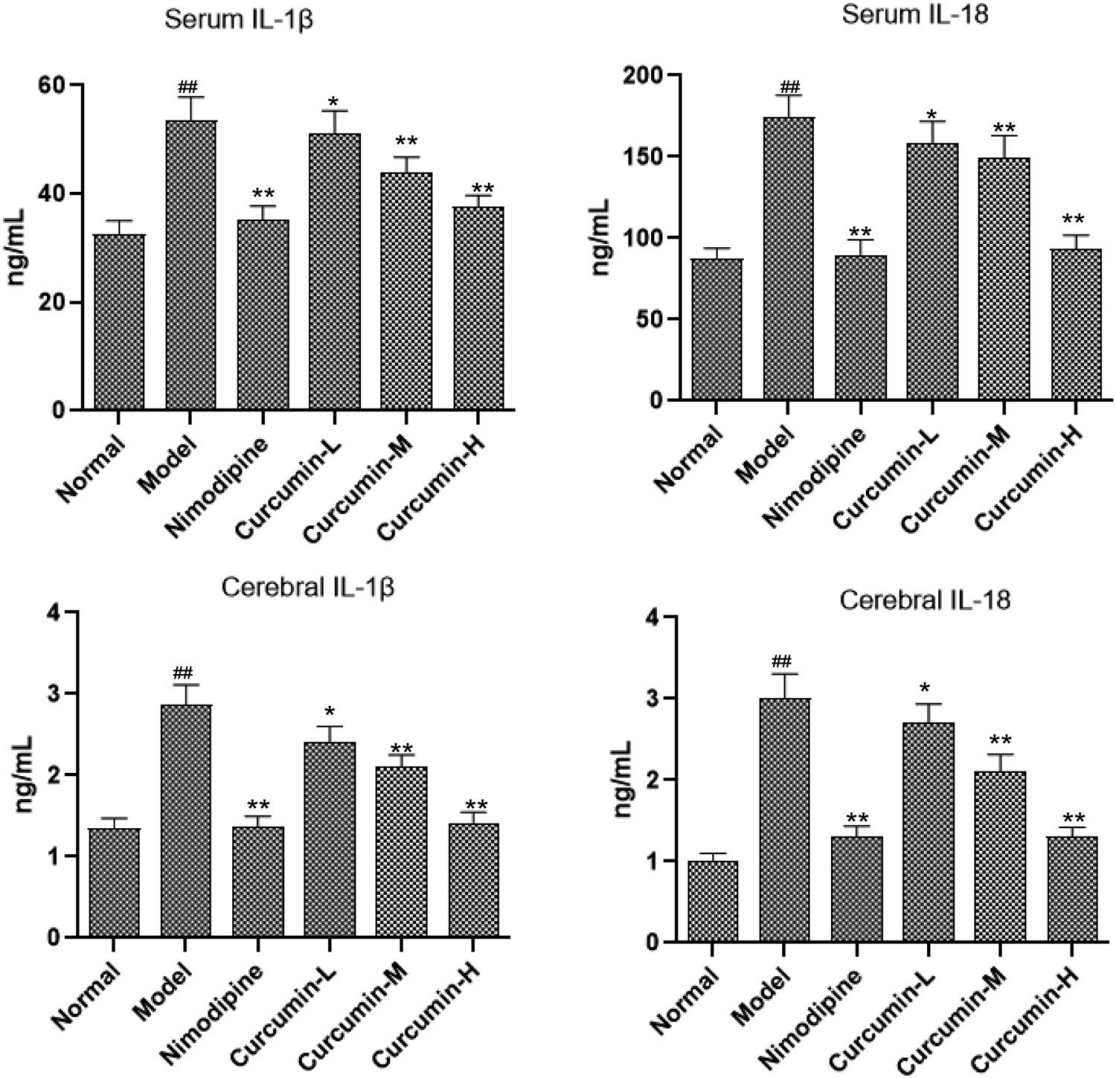
Effect of curcumin on the content of IL-1β and IL-18 in serum and brain tissue of rats. ELISA was performed to analyse IL-1β and IL-18 levels. Compared with the normal, ^##^*p* < 0.01; compared with the model, *p < 0.05, **p < 0.01.

It is reported that CHOP–caspase-11 could induce the release of IL-1β and IL-18 in ischaemic reperfusion (Liu et al. 2018). Thus, in this study, ELISA was used to detect inflammatory factors IL-1β and IL-18 levels in the serum and brain tissue. Compared with the normal control group, IL-1β and IL-18 levels in the serum and brain tissue of the model group increased significantly (*p* < 0.01) (Figure 5). The positive control group and the medium and high dose curcumin groups had significantly reduced levels of IL-1β and IL-18 than model group (*p* < 0.01). Thus, curcumin may inhibit the neuroinflammation after cerebral ischaemia and reperfusion.

### Effect of curcumin on the expression of ERK, CHOP and caspase-11 mRNA in the brain tissues of rats

**Figure 6.**
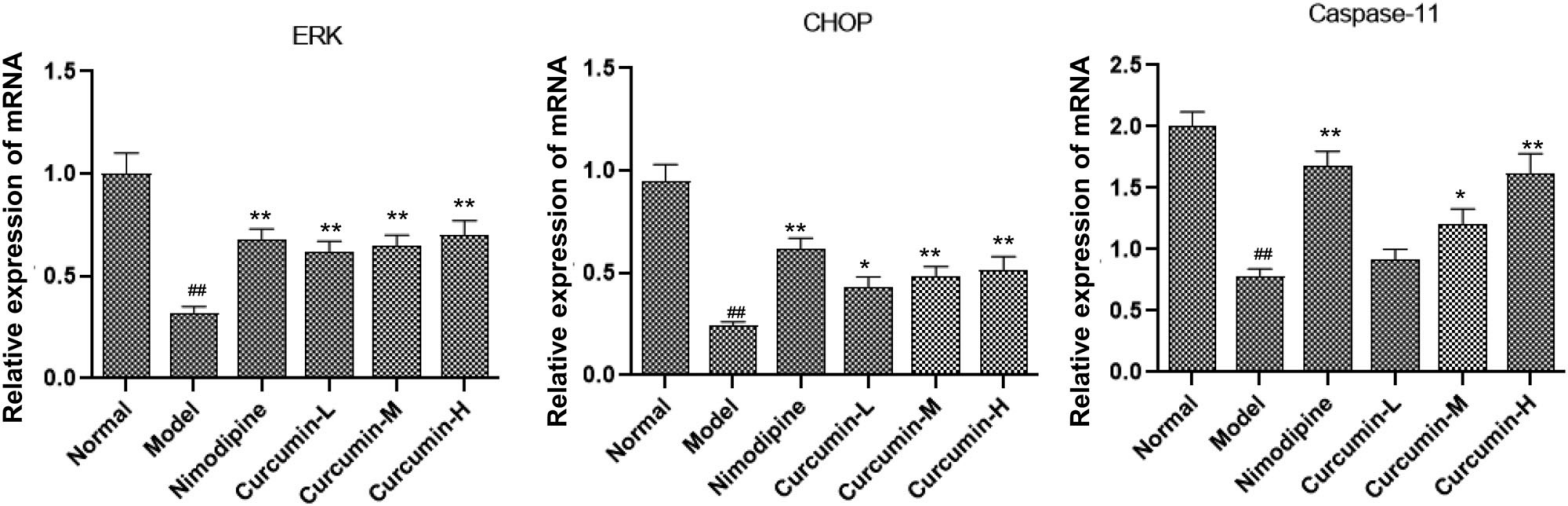
Effect of curcumin on the expression of *ERK*, *CHOP* and *caspase-11* mRNA in rat brain tissue. Real-time PCR was performed to detect *ERK*, *CHOP* and *caspase-11* mRNA levels. Compared with the normal, ^##^*p* < 0.01; compared with the model, *p < 0.05, **p < 0.01.

The *ERK*, *CHOP* and *caspase-11* mRNA levels were assessed with real-time PCR. As shown in Figure 6, the model group had significantly lower *ERK*, *CHOP* and *caspase-11* mRNA levels than normal control group (*p* < 0.01). However, these levels significantly increased in positive control group and curcumin group (medium and high doses) than model group (*p* < 0.01).

### Effect of curcumin on the protein content of p-ERK, p-CHOP and caspase-11 in the brain tissue of rats

**Figure 7.**
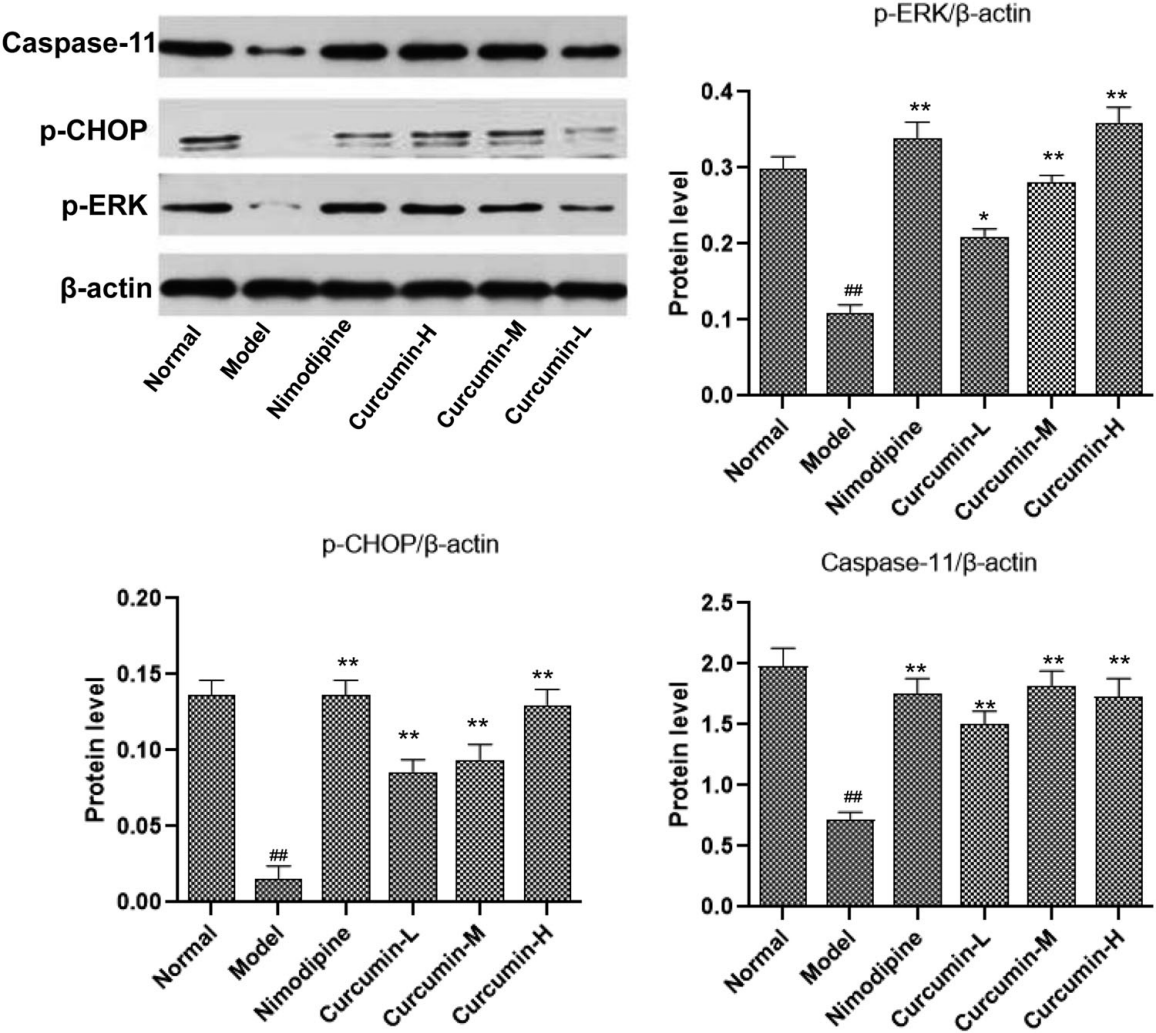
Effect of curcumin on the expression of ERK, CHOP and caspase-11 protein in rat brain tissue. Western blot was conducted to detect ERK, CHOP and caspase-11 protein levels. Compared with the normal, ^##^*p* < 0.01; compared with the model, *p < 0.05, **p < 0.01. Lane A: normal; lane B: model; lane C: nimodipine; lane D: curcumin-H; lane E: curcumin-M; lane F: curcumin-L.

Western blot was used to detect the protein levels of p-ERK, p-CHOP and caspase-11 in the brain tissues (Figure 7). Consistent with the mRNA results, the cerebral ischaemia and reperfusion injury in the model group significantly reduced the protein content of p-ERK, p-CHOP and caspase-11 in the brain tissues (*p* < 0.01). This reduce was reversed by treatment with nimodipine (positive control) and curcumin (medium and high doses) (*p* < 0.01).

## Discussion

Cerebral ischaemia and reperfusion can easily cause serious damage to important organs such as the brain, heart, lungs and kidneys. It is one of the most common causes of death and disability in the world (Li et al. 2019). The pathophysiological mechanism of CIRI is very complicated, which involves the interaction of multiple mechanisms, including neuroinflammation, oxidative stress, oxygen free radical damage and intracellular calcium overload. These factors may directly or indirectly lead to the apoptosis of brain nerve cells, and further cause the neurological dysfunction (Zhang and Wang 2014). At present, typical drugs for the treatment of CIRI include neuroprotective agents, antioxidants, anti-apoptotic drugs and anti-inflammatory drugs (Zhao et al. 2015; Ai et al. 2017). Curcumin has many functions such as antioxidation, anti-coagulation, anti-inflammation, scavenging free radicals, anti-atherosclerosis and inhibiting tumour growth (Giordano and Tommonaro 2019; Patel et al. 2020). It also has certain effects on cardiovascular diseases (Liu et al. 2012). The results of this study found that the levels of inflammatory factors IL-1β and IL-18 in the serum and brain tissue of rats after administration of curcumin were significantly lower than those in the model group. In addition, curcumin also improved the neurobehavioural score of rats and improved the neurological damage of rats.

Caspase-11 is a protein with a dual role that can cause apoptosis and inflammation (Vanaja et al. 2016; Lu et al. 2019). CHOP, also known as growth arrest and DNA damage-inducing gene 153, is one of the transcription factors of endoplasmic reticulum stress, which can cause cell apoptosis. Cerebral ischaemia and reperfusion cause abnormal endoplasmic reticulum stress response and inhibit the expression of CHOP (Lv et al. 2018; Song and Ping 2018). Meanwhile, it will activate the related signal pathway of inflammatory response, leading to the overexpression of inflammatory factor IL-1β (Lv et al. 2018; Song and Ping 2018). Consistently, this study found that after curcumin administration, the protein and mRNA expression of caspase-11 and CHOP increased, while the expression of the inflammatory factor IL-1β decreased, suggesting that curcumin may inhibit apoptosis and inflammation via caspase-11 and CHOP. ERK1/2 is one of the important members of the MAPK family. The ERK1/2 signal transduction pathway can promote cell proliferation and differentiation, inhibit apoptosis and reduce tissue inflammation after being activated by external stimuli. ERK activated by phosphorylation can prevent nerve functional defects and neuronal death and have a protective effect on cerebral ischaemia and reperfusion (Feng and Hu 2018; Yuan et al. 2018; Zhao et al. 2018). Similarly, we showed that the protein level of p-ERK in the brain tissue of rats increased significantly after curcumin administration, indicating that curcumin may exert its effects through regulating ERK.

## Conclusions

Curcumin can inhibit neuronal apoptosis in rats with CIRI and reduce their neuroinflammatory response in rats. This effect may be acted through caspase-11, CHOP and ERK. Our findings may further reveal the pathological mechanism of CIRI and support the use of curcumin as potential drug for the treatment of CIRI.
